# Novel Fabrication of Basalt Nanosheets with Ultrahigh Aspect Ratios Toward Enhanced Mechanical and Dielectric Properties of Aramid Nanofiber‐Based Composite Nanopapers

**DOI:** 10.1002/advs.202302371

**Published:** 2023-07-23

**Authors:** Dexian Ji, Shunxi Song, Yuming Lyu, Wei Ren, Linghao Li, Bin Yang, Meiyun Zhang

**Affiliations:** ^1^ Key Laboratory of Auxiliary Chemistry and Technology for Chemical Industry Ministry of Education Shaanxi Province Key Laboratory of papermaking Technology and Specialty paper Development College of Bioresources Chemical and Materials Engineering Shaanxi University of Science & Technology Xi'an 710021 P. R. China; ^2^ Shaanxi Collaborative Innovation Center of Industrial Auxiliary Chemistry and Technology Shaanxi University of Science and Technology Xi'an 710021 P. R. China

**Keywords:** aramid nanofibers, basalt nanosheets, composite nanopapers, electrical insulation, mechanical strength

## Abstract

The rapid development of modern electrical equipment has led to urgent demands for electrical insulating materials with mechanical reliability and excellent dielectric properties. Herein, basalt nanosheets (BSNs) with high aspect ratios (≈780.1) are first exfoliated from basalt scales (BS) through a reliable chemical/mechanical approach. Meanwhile, inspired by the layered architecture of natural nacre, nacre‐mimetic composite nanopapers are reported containing a 3D aramid nanofibers (ANF) framework as a matrix and BSNs as ideal building blocks through vacuum‐assisted filtration. The as‐prepared ANF‐BSNs composite nanopapers exhibit considerably enhanced mechanical properties with ultralow BSNs content. These superiorities are wonderfully integrated with exceptional dielectric breakdown strength, prominent volume resistivity, and extremely low dielectric constant and loss, which are far superior to conventional nacre‐mimetic composite nanopapers. Notably, the tensile strength and breakdown strength of ANF‐BSNs composite nanopapers with a mere 1.0 wt% BSNs reach 269.40 MPa and 77.91 kV mm^−1^, respectively, representing an 87% and 133% increase compared to those of the control ANF nanopaper. Their properties are superior to those of previously reported nacre‐mimetic composite nanopapers and commercial insulating micropapers, indicating that ANF‐BSNs composite nanopapers are a highly promising electrical insulating material for miniaturized high‐power electrical equipment.

## Graphical abstract

Basalt nanosheets (BSNs) with high aspect ratios are first exfoliated from basalt scales (BS) through a reliable chemical/mechanical approach. Meanwhile, the nacre‐inspired concept is employed to develop ANF‐BSNs composite nanopapers containing a 3D ANF framework as a matrix through a vacuum‐assisted filtration method. The bioinspired ANF‐BSNs composite nanopapers exhibit considerably enhanced mechanical and electrical insulating properties with ultralow BSNs content.

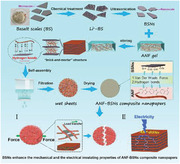

## 1 Introduction

The rapid development of rail transit, aerospace, and other fields has put forward higher requirements for electrical insulating materials, whose reliability depends on many factors, including electrical insulating performance, thermal stability, mechanical properties, aging, etc.^[^ ^1^ ^]^ Among these reported high‐performance electrical insulating materials, aramid nanofibers (ANF)‐based films or papers are desired as electrical insulating materials and have been extensively utilized in high‐voltage power equipment, high‐speed trains, and airplanes,^[^ ^2^, ^3^, ^4^ ^]^ owing to their exceptional insulating and mechanical properties, as well as their favorable thermal stability.^[^ ^5^, ^6^ ^]^ Nevertheless, the presence of bubbles, cracking, deformation, and aging problems in ANF‐based films or papers significantly weakened their electrical insulating properties, resulting in frequent occurrences of partial discharge or breakdown.^[^ ^7^ ^]^ Numerous nano‐fillers (such as carbon nitride nanosheets (CNNs),^[^ ^7^, ^8^ ^]^ boron nitride nanosheets (BNNs),^[^ ^9^ ^]^ montmorillonite,^[^ ^10^ ^]^ etc.) have been reported to improve the structure of the ANF‐based films or papers, thereby enhancing their electrical insulating properties.

Recently, silicate minerals (such as mica, vermiculite, rectorite, etc.) have a wide range of applications in various fields of electrical insulation,^[^ ^11^ ^]^ mechanical reinforcement,^[^ ^12^ ^]^ fire protection,^[^ ^13^ ^]^ and flexible devices,^[^ ^14^ ^]^ owing to their intriguing mechanical and physical properties.^[^ ^15^ ^]^ Notably, basalt scales (BS), one of the most abundant silicate minerals, demonstrate excellent thermal and chemical stability, as well as sound absorption, especially in mechanical and electrical insulating properties, due to their distinctive chemical composition and structure.^[^ ^16^, ^17^, ^18^, ^19^, ^20^ ^]^ Nevertheless, BS have not been utilized in the electrical insulating field despite their outstanding physical and chemical properties. Assembling BS with ANF to fabricate the composite micropapers may be a promising way to fully realize their inherent potential. Unfortunately, it is hard to form a favorable interface combination between ANF and BS due to the large size and chemical inertness of BS. Therefore, the composite papers that incorporate ANF and BS exhibit a significantly low tensile strength, thereby restricting their practical application. Generally, the biomimetic assembly of superior quality nanosheets into nacre‐mimetic “brick‐and‐mortar” structures is an effective approach to fabricate high‐performance macroscopic papers.^[^ ^21^ ^]^

Several exfoliation methods, such as in‐situ polymerization, melt intercalation, and solution intercalation, have been efficaciously devised for the fabrication of silicate nanosheets (such as mica nanosheets,^[^ ^22^, ^23^ ^]^ vermiculite nanosheets,^[^ ^24^ ^]^ and rectorite nanosheets^[^ ^10^ ^]^). Nevertheless, the crystal structure of BS is prone to being destroyed and forming an amorphous structure due to the high molding process temperature exceeding 1300 °C.^[^ ^19^, ^25^ ^]^ Besides, the polyhedral structures (such as silico‐oxygen tetrahedron, alumino‐oxygen tetrahedron, alumino‐oxygen octahedron, etc.) are interconnected to form an unbounded 3D lattice via co‐edge or co‐top within the internal framework of BS.^[^ ^26^ ^]^ Because of the cationic isocrystalline substitution within these structural units, an excess of negative charges is produced. To maintain electrical neutrality within the internal system, the lateral area of the structural units in BS is compensated by cations (such as Ca^2+^, K^+^, Na^+^, etc.) and other metal ions. The aforementioned facts indicate that BS, lacking a layered structure like other 3D‐layered crystals, is obviously unsuitable for conventional liquid exfoliation methods.^[^ ^27^, ^28^, ^29^ ^]^ Consequently, it would be of great significance to develop a simple and inexpensive exfoliation method to produce basalt nanosheets (BSNs) with high aspect ratios and assembled with ANF into large‐size composite nanopapers with similar or superior performance relative to other silicate nanosheets.

Herein, we first develop a novel and facile exfoliating method to obtain superior quality 2D BSNs with high aspect ratios (≈780.1) from BS. The obtained BSNs suspension exhibits excellent stability and inherits the excellent properties of the original BS. Subsequently, the nacre‐inspired concept was employed to develop a type of ANF‐BSNs composite nanopaper containing a 3D ANF framework as a matrix through a vacuum‐assisted filtration method. The bioinspired ANF‐BSNs composite nanopapers demonstrate a nacre‐mimetic layered structure where oriented BSNs are embedded into a 3D ANF framework through hydrogen bonding. Furthermore, the resulting ANF‐BSNs composite nanopapers with an ultralow BSNs content of 1.0 wt% (denoted as ANF‐BSNs (1.0 wt%)) exhibit exceptional mechanical properties, including a tensile strength of 269.40 MPa, a modulus of 4.08 GPa, and a toughness of 16.22 MJ m^−3^. Simultaneously, these composite nanopapers exhibit significantly enhanced dielectric breakdown strength, exceptional flexibility, prominent volume resistivity, and extremely low dielectric constant and loss. These properties are better than those of the control ANF nanopapers, numerous previously reported ANF‐based nanopapers, and commercial insulating papers, rendering them a highly promising electrical insulating material for practical applications.

## 2 Results and Discussion

### 2.1 Fabrication and Structural Characteristics of Basalt Nanosheets (BSNs)

In order to satisfy the requirements of high‐performance ANF‐based electrical insulating papers, it is of great significance to fabricate BSNs with high aspect ratios. In this work, BSNs were fabricated by using pure BS as the raw material. BS exhibit a smooth and dark gray flake‐like morphology, and the majority of BS are 100–180 µm in lateral size and 2–3 µm in thickness. Additionally, numerous metal ions exist in the internal BS (Figure S1 and Table S1, Supporting Information).

**Figure 1:**
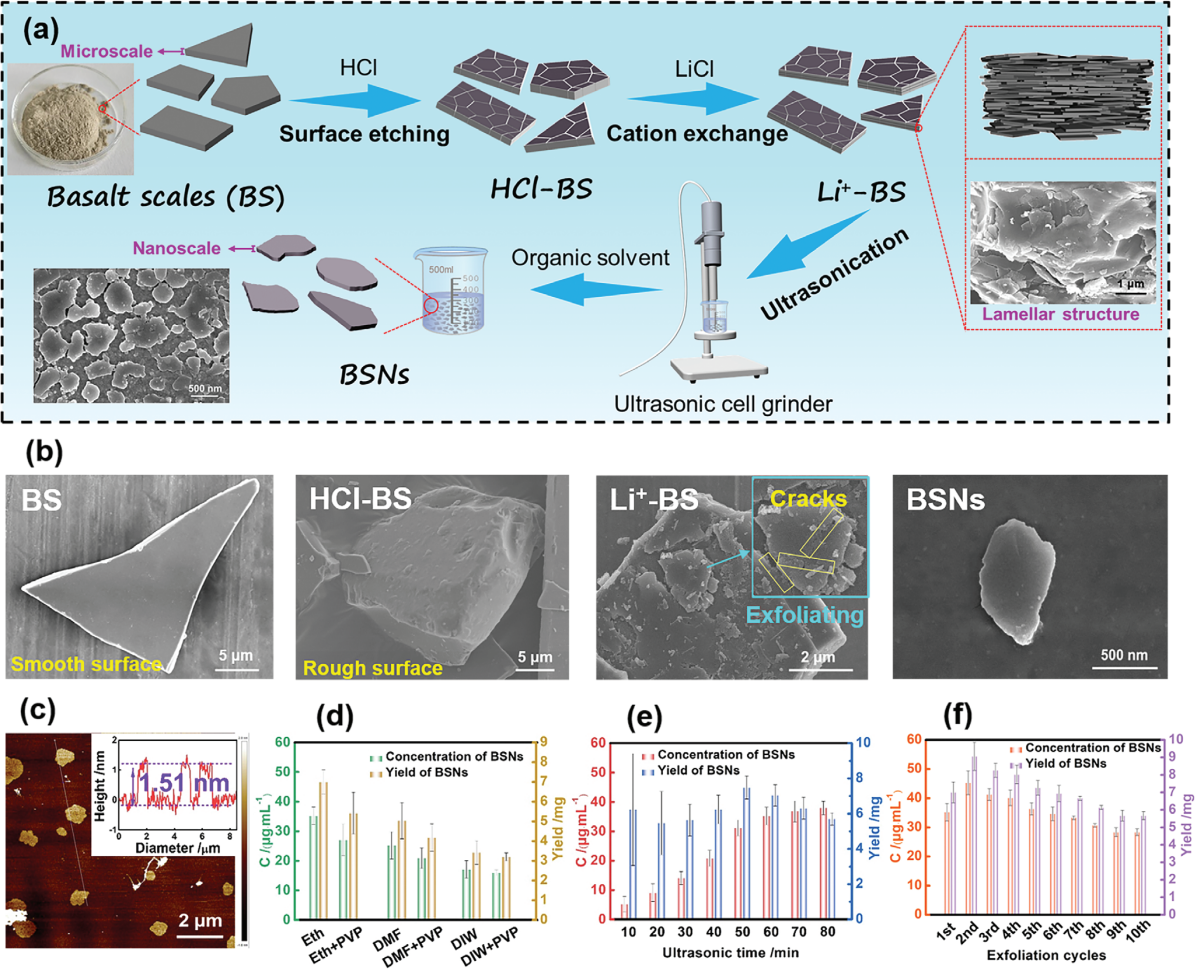
Fabrication of BSNs. a) Schematic illustration of the fabrication process of BSN; b) Scanning electron microscopy (SEM) images of BS, HCl‐BS, Li^+^‐BS, and BSNs; c) Atomic force microscopy (AFM) image and height curve of BSNs; d) The concentrations and yield of the BSNs dispersions prepared by the ultrasonication of Li^+^‐BS with different solvents (1 h for each exfoliation process, 1 cycle); e) The concentrations and yield of BSNs dispersions obtained by different ultrasonic times; f) The concentrations and yield of BSNs dispersions obtained by different cycle times of Li+‐BS and ethanol mixture (1 h for each exfoliation process, 10 cycles).

The detailed exfoliation process is illustrated in **Figure** **1**a. The original BS were activated through Hydrochloric acid (HCl) treatment (the obtained material was denoted as HCl‐BS) to dissolve the metal oxide in their internal structure. The surface roughness (Ra) of HCl‐BS increases significantly from 1.67 nm of BS to 20.87 nm (Figure S2a, Supporting Information). Subsequently, Li ions were introduced into the inner structure of activated BS through ion exchange, replacing the alkali metal ions with large diameters to obtain BS modified by the lithium ion exchange reaction (the obtained material was denoted as Li^+^‐BS). These Li ions would combine with H_2_O to form hydrated ions with a larger diameter, facilitating the expansion of the inner spacing of Li^+^‐BS and resulting in irregular the inner and exterior fractures of the original structure.^[^ ^30^ ^]^ The maximum cation exchange capacity (CEC) occurred in Li^+^‐BS, and the maximal CEC was 22.79 mmol g^−1^, which increased by 136% compared to that of the control BS (9.64 mmol g^−1^) (Table S2, Supporting Information). This result verified the successful ion exchange process for BS. In addition, numerous lamellae were produced and stacked together, retaining the original scale structure of BS from an overall perspective (Figure 1b). After ultrasonication was applied, the cavitation effectively disrupted the weak interactions (van der Waals forces) between the irregular lamellae, facilitating the exfoliation of Li^+^‐BS into BSNs.^[^ ^23^ ^]^ The successful exfoliation of BSNs is confirmed by transmission electron microscopy (TEM) images (Figure S2b, Supporting Information). The size of the obtained BSNs ranges from 0.8 to 2.0 µm (Figure 1b; Figure S2c, Supporting Information), and their thickness is ≈1.51 nm (Figure 1c). Furthermore, BSNs exhibit an extremely low Zeta potential of −42.837 mV, indicating a homogeneous dispersion of BSNs suspension. The perfect dispersion is further confirmed by the Tyndall effect of BSNs (Figure S2d, Supporting Information).

The concentration and yield of the BSNs were found to be related to the type of dispersion solvent, the ultrasonic time, and the reaction process. According to previous reports,^[^ ^10^, ^31^ ^]^ anhydrous ethanol (Eth) and N,N‐Dimethylformamide (DMF) aqueous solutions were generally utilized to dissolve organic and inorganic compounds. Hence, we investigated the efficiency of exfoliation from BS to BSNs in the above dispersant. Normally, polyvinyl pyrrolidone (PVP) is always utilized as an intercalator in the preparation of silicate nanosheets, because PVP can enter the interlayer due to the hydrogen bonding interactions between the carbonyl groups of PVP and the silicon hydroxyl groups of silicate compounds.^[^ ^10^ ^]^ Herein, PVP was added separately to anhydrous ethanol, DMF, and Deionized water (DIW), respectively, aiming to improve the preparation efficiency. Impressively, the BSNs dispersion with PVP as the intercalation agent shows the lowest concentration and yield of BSNs (Figure 1d; Figure S3a and Table S3, Supporting Information). It is mainly because the presence of PVP leads to the aggregation of Li^+^‐BS through hydrogen‐bonding interactions, thereby mitigating the effects of the ultrasonic cavitation impact on the exfoliation process. When a larger volume (250 mL) dispersion is treated under 800 W ultrasonication power, an acceptable production rate of BSNs with ≈7.0 mg h^−1^ can be easily achieved (Figure S4 and Table S3, Supporting Information). In addition, the yield of the BSNs in different dispersion solvents, ultrasonic times, and reaction processes was also studied. BSNs dispersion in pure anhydrous ethanol shows the highest concentration of 35.0 µg mL^−1^ and yield of 7.0 mg (prepared by 1 h ultrasonication) compared to dispersion in the other five dispersion solvents, partly due to the similar surface energy between BSNs and anhydrous ethanol (Table S3, Supporting Information). Additionally, the concentration of BSNs gradually increases with increasing ultrasonic time. However, it is observed that the increasing rate starts to diminish after 60 min (Figure 1e; Figure S3b and Table S4, Supporting Information). Typically, the preparation process of nanosheets (such as mica nanosheets, BNNs, CNNs, etc.) is a single process.^[^ ^7^, ^8^, ^9^, ^10^, ^11^ ^]^ In order to increase the yield of BSNs, Li^+^‐BS was treated repeatedly with ultrasonication process. In the typical experiment, the upper suspension obtained after the ultrasonication process was collected, while the sediment of Li^+^‐BS was reserved for subsequent ultrasonication treatment. This procedure was repeated for 10 cycles. It can be observed that the concentration of BSNs gradually decreased with an increase in the number of ultrasonication cycles, partly because the lamellae structure on Li^+^‐BS was finite and gradually exfoliated by ultrasonication (Figure 1f; Figure S3c and Table S5, Supporting Information). However, the yield of 70.8 mg is attained in the whole process (10 cycles), which is significantly higher than the yield obtained in the process containing only the first ultrasonication (Table S5, Supporting Information).

**Figure 2:**
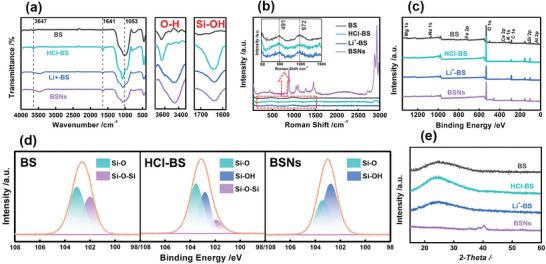
Chemical structure characterization of BSNs. a) FTIR spectra, b) Raman scattering, c) XPS spectra of BS, HCl‐BS, Li^+^‐BS, and BSNs; d) XPS Si2p of BS, HCl‐BS, and BSNs; e) XRD patterns of BS, HCl‐BS, Li^+^‐BS, and BSNs.

To demonstrate the successful fabrication of BSNs, Fourier transform infrared (FTIR) spectra, Raman scattering, X‐ray photoelectron spectroscopy (XPS) spectra, and X‐ray diffraction (XRD) measurements were employed. The formation process of BSNs is observed in FTIR spectra. As displayed in **Figure** **2**a, the absorption peak at 3647 cm^−1^ of BS and HCl‐BS is assigned to the stretching vibration of ─OH bond.^[^ ^32^ ^]^ Significantly, the characteristic peak of the ─OH group for Li^+^‐BS and BSNs shifts from 3647 to 3468 cm^−1^. The absorption peak at 1643 cm^−1^ is attributed to Si─OH, the wider vibration peak between 850 and 1274 cm^−1^ is assigned to the symmetric and asymmetric stretching vibrations of Si─O─Si.^[^ ^19^ ^]^ Notably, the Si─OH peak appears in the spectra of HCl‐BS, Li^+^‐BS, and BSNs, indicating that the surface etching is conductive for the activation of BS and the exposure of functional groups on the surface of basalt material. The ─OH stretching peak exhibits a distinct blueshift in Li^+^‐BS and BSNs compared to BS and HCl‐BS. Specifically, the blue shift from 1643 cm^−1^ (─Si─OH groups of HCl‐BS) to 1637 cm^−1^ (─Si─OH groups of Li^+^‐BS and BSNs) is also observed in the FTIR spectra. This result implies that the surface lithium atom forms a hydrogen bond with ─Si─OH group in Li^+^‐BS and BSNs.^[^ ^11^ ^]^ However, these shifts are relatively slight, probably due to the formation of hydrogen bonds between lamellae in Li^+^‐BS and BSNs.

To further explore the formation mechanism of BSNs, Roman scattering was employed to characterize the internal structure of basalt material. The result, as shown in Figure 2b, reveals that the characteristic peak at 491 cm^−1^ is the typical peak of BS, HCl‐BS, and Li^+^‐BS and corresponds to the bending vibration of Si‐O_br_‐Si. Additionally, the peak at 972 cm^−1^ is attributed to the Si─O_nb_ stretching vibration in the structural unit with a non‐bridging oxygen number of two, implying the presence of a chain‐like [Si_2_O_6_]^−^ structure. Notably, more new peaks are observed in the spectra of BSNs. The peak at 434 cm^−1^ is assigned to the M─O vibrations and the bending vibrations of Si─O─Si(Al). Furthermore, the peak at 1051 cm^−1^ is attributed to the asymmetric stretching vibration of Si(Al)‐O_nb_, with a non‐bridging oxygen number of one in the structural unit of T_2_O_5_ (such as [Si_2_O_5_]^2−^),^[^ ^33^ ^]^ which verified the presence of lamellar structure in BSNs.

The surface chemical properties of BSNs have a significant impact on the performance of the subsequent composite nanopapers by influencing their internal combination with ANF. Compared to BS, the elemental types and content in HCl‐BS and Li^+^‐BS are found to decrease. This is mainly attributed to the dissolution of metal oxides and the cation‐exchange reaction of metal elements^[^ ^34^ ^]^ (Figure 2c), which is also confirmed by X‐ray Fluorescence (XRF) results (Table S1, Supporting Information). C 1s is found in the XPS spectra of all samples, which is probably attributed to the adsorption of CO_2_ before the XPS analysis (Figure 2d; Figure S5, Supporting Information). It can be observed in the Si 2p spectrum that the peaks at 103.44 and 101.74 eV of the above samples correspond to Si─O and Si─O─Si bonds. Additionally, a new peak at 102.52 eV is observed in the Si 2p spectrum of HCl‐BS, Li^+^‐BS, and BSNs, which is assigned to Si─OH^[^ ^19^, ^35^ ^]^ (Figure 2d; Figure S6, Supporting Information). This result is consistent with the FTIR analysis (Figure 2a). Notably, the Si─O─Si peak is absent in the XPS spectra of Li^+^‐BS and BSNs. This can be attributed to the significant swelling of BS when larger radius hydrated cations (Li^+^) enter BS through cation‐exchange interaction, resulting in the lamellar bulging and cracking. Furthermore, the cation‐exchange interaction increases the probability of proton attack on the lamellar structure, leading to the decomposition of the BS lamellar,^[^ ^36^ ^]^ finally resulting in the break of Si─O─Si bonds in Li^+^‐BS.

The XRD patterns, illustrated in Figure 2e, demonstrate that only a broad peak ranging from 20° to 30° can be observed in the XRD patterns of BS, HCl‐BS, and Li^+^‐BS. This result indicates that these basalt materials retain their amorphous structure (proximally ordered and remotely disordered structure), which may be attributed to the fabrication process of BS from basalt ore.

### 2.2 Fabrication and Characterizations of ANF‐Based Composite Nanopapers

**Figure 3:**
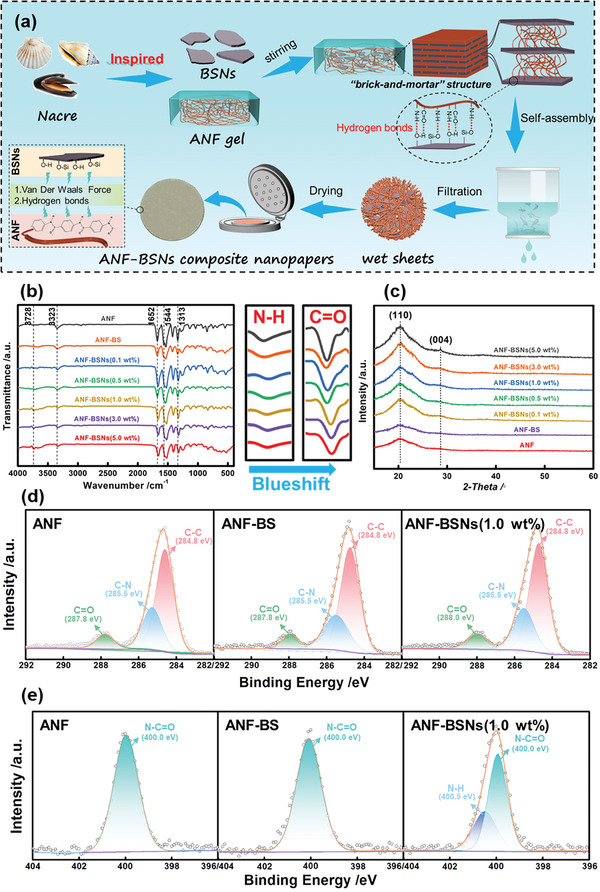
Fabrication and interfacial structure characterization of ANF‐BSNs composite nanopapers. a) Schematic illustration of the fabrication process of ANF‐BSNs composite nanopapers; b) The FTIR spectra, c) XRD patterns, d) XPS C 1s, and e) N 1s spectra of ANF nanopapers, ANF‐BS micropapers with 1.0 wt% BS, and ANF‐BSNs composite nanopapers with 1.0 wt% BSNs.

The rapid development of modern electrical equipment toward miniaturization and high power makes urgent demands on the mechanical properties, electrical insulating performance, and high‐temperature resistance of electrical insulating materials.^[^ ^37^, ^38^, ^39^ ^]^ The obtained BSNs demonstrate great potential for fabricating electrical insulating nanopapers due to their remarkable mechanical and electrically insulating properties. However, due to the weak interfacial interaction among BSNs, it is hard for them to form films or papers. To solve this limitation, the concept of composite nanopapers is proposed. ANF, possessing a large specific area and substantial crystallinity, not only retains the mechanical and electrical insulating properties of aramid microfibers but also exhibits strong interaction capabilities with other phases owing to the abundant amide groups on their surface^[^ ^40^ ^]^ (Figure S7, Supporting Information). Therefore, inspired by the layered “brick‐and‐mortar” structure of natural nacre, BSNs were co‐assembled with ANF into nacre‐mimetic ANF‐BSNs composite nanopapers. These composite nanopapers in this work are synthesized through a vacuum‐assisted filtration method (**Figure** **3**a). During the vacuum‐assisted filtration process, as the dispersion was filtered through the membrane, some nanofibers might initially be filtered as a fibriform membrane. Nevertheless, this process would be quickly hindered due to the deposition of BSNs. The deposited BSNs with high aspect ratios on the membrane surface restrict the further loss of nanofibers, resulting in a uniform deposition of both BSNs and ANF in the wet sheet fabrication process. Eventually, the layered “brick‐and‐mortar” structure can be formed as the thickness of the wet sheets increases, wherein 2D BSNs and ANF deposit on top of each other.

As shown in Figure 3b, ANF nanopapers, ANF‐BS micropapers with 1.0 wt% BS (denoted as ANF‐BS), and ANF‐BSNs composite nanopapers with 1.0 wt% BSNs (denoted as ANF‐BSNs (1.0 wt%)) display characteristic peaks at 3323 cm^−1^, corresponding to the stretching vibration of N─H. Additionally, the peak at 1652 cm^−1^ is mainly attributed to the C═O stretching in the amide unit. However, in the enlarged FTIR spectra, the peak positions of the symmetrical stretching vibration bonds of N─H and C═O exhibit a continuous blueshift as the BSNs mass fraction increases, indicating the formation of hydrogen‐bonding between BSNs and ANF.^[^ ^11^ ^]^ As displayed in Figure 3c, the XRD pattern for ANF exhibits broad peaks at 20.5°and 28.0°, corresponding to (110) and (004) crystal planes, respectively. The diffraction peaks of ANF‐BSNs composite nanopapers become sharper and more intense as the BSNs mass fraction increases, partly due to the formation of hydrogen bonding.^[^ ^19^ ^]^ The XPS wide‐scan spectra and the high‐resolution spectra of C 1s and N 1s of ANF nanopapers, ANF‐BS micropapers, and ANF‐BSNs composite nanopapers are further discussed (Figure 3d; Figure S8a, Supporting Information). The C 1s core‐level photoelectron spectra of ANF nanopapers and ANF‐BS micropapers could be curved into five peak components located at 284.8, 285.5, and 287.8 eV, which are attributed to C─C, C─N, and C═O, respectively. Notably, the peak attributed to C═O shifts to a higher binding energy of 288.0 eV in the XPS spectrum of C 1s for ANF‐BSNs composite nanopapers. Furthermore, the spectra of N 1s for ANF nanopapers and ANF‐BS micropapers exhibit a single binding energy peak at ≈400.0 eV. However, in the spectra of N 1s for ANF‐BSNs composite nanopapers, the peak can be decomposed into two components, including a higher binding energy peak at ≈400.5 eV. The results indicate that the chemical environment of C═O and N─H has been changed, further demonstrating the formation of hydrogen‐bonding interactions between BSNs and ANF.^[^ ^41^, ^42^, ^43^ ^]^ Moreover, the loading content of BSNs in ANF‐BSNs composite nanopapers is calculated to be 0.935 wt% based on the TG curves^[^ ^44^ ^]^ (Figure S8b, Supporting Information), which is close to the addition of BSNs (1.0 wt%) in the fabrication of ANF‐BSNs composite nanopapers.

**Figure 4:**
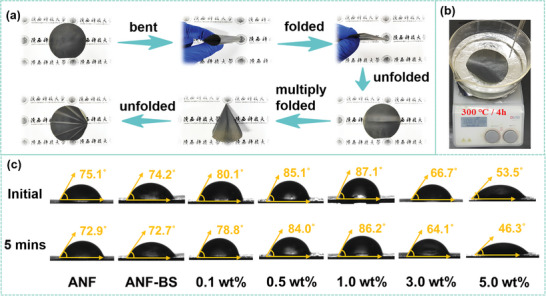
The ANF‐BSNs composite nanopapers with excellent comprehensive performances. a) The photographs of ANF‐BSNs composite nanopapers to show their excellent flexibility; b) The photographs of ANF‐BSNs composite nanopapers in silicone oil‐bath environment at 300 °C for 4 h; c) The contact angle of ANF nanopapers, ANF‐BS micropapers, and ANF‐BSNs composite nanopapers.

As illustrated in **Figure** **4**a, ANF‐BSNs composite nanopapers exhibit excellent bendability and foldability. In the conventional sense, it is logical that ANF nanopapers are used in the insulation field. Notably, it could be seen that the overall appearance of ANF‐BSNs composite nanopapers remains intact even after exposure to a silicone oil‐bath environment at a supremely high temperature, confirming their use in such extreme environments (Figure 4b). Additionally, ANF‐BSNs composite nanopapers with 1.0 wt% BSNs demonstrate slightly higher hydrophobicity (87.1°) and water resistance (decrease only 0.9° after 5 min) (Figure 4c), since the nacre‐mimetic layered structure in ANF‐BSNs composite nanopapers with 1.0 wt% BSNs effectively blocks the penetration of water molecules. However, the contact angle decreases in ANF‐BSNs composite nanopapers containing 3.0 wt% BSNs and 5.0 wt% BSNs. This is reasonable because the excess addition of the BSNs leads to their non‐uniform distribution, resulting in interfacial and internal defects for water molecules.

### 2.3 Mechanical Characterizations of ANF‐Based Composite Nanopapers

Electrical insulating films or papers are prone to various types of damage, such as mechanical damage and dielectric aging, during their service careers, resulting in an undoubtedly decreased transformer life.^[^ ^45^ ^]^ Consequently, the performance of insulating films or papers, particularly their mechanical properties and dielectric strength, is extremely crucial to the service life of electrical apparatuses.^[^ ^10^ ^]^

**Figure 5:**
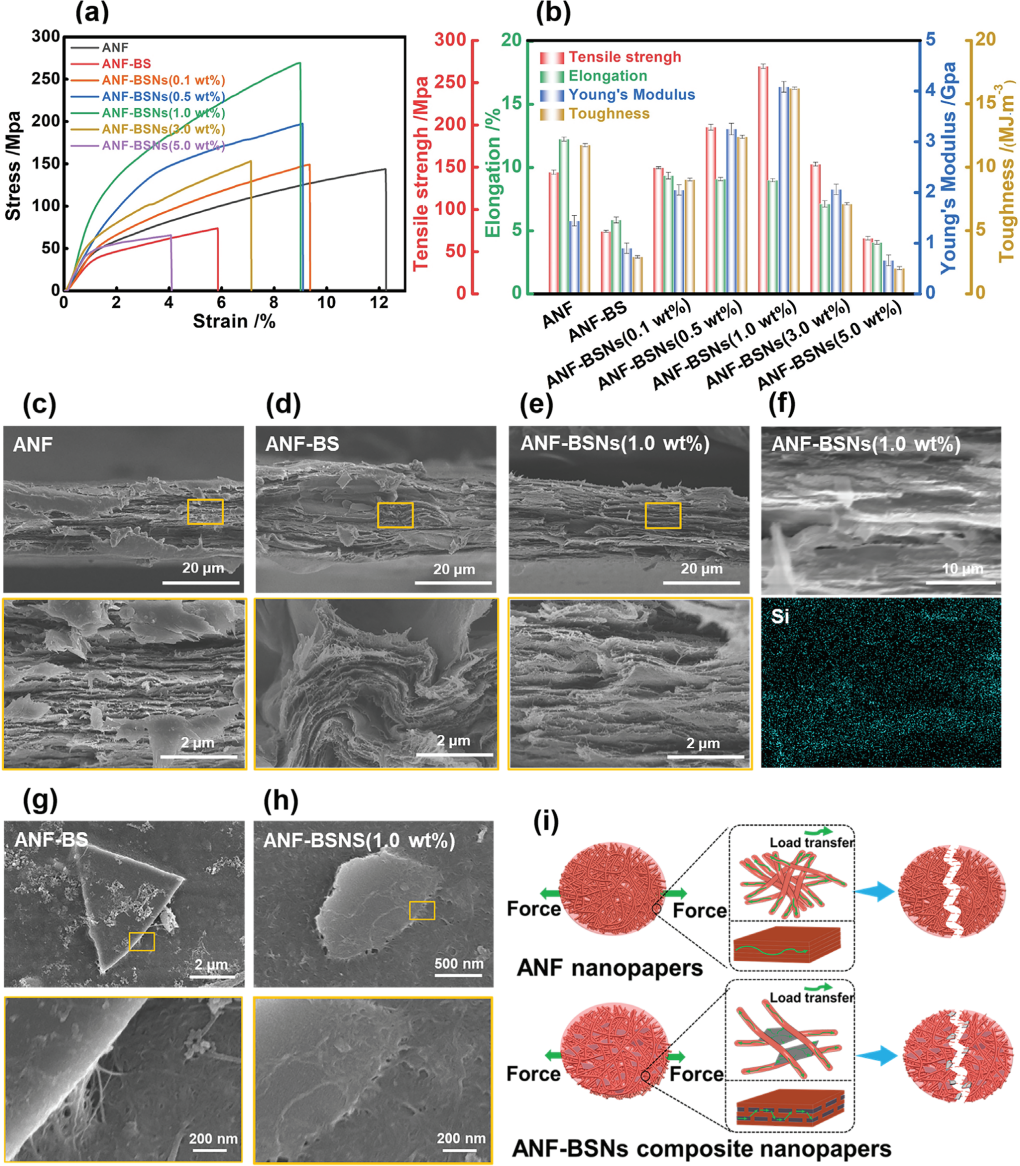
The mechanical properties of ANF‐BSNs composite nanopapers. a) Tensile mechanical properties of the ANF‐based composite nanopapers. b) Stress–strain curves; tensile strength, elongation, modulus, and toughness of ANF nanopapers, ANF‐BS micropapers, and ANF‐BSNs composite nanopapers. c) The tensile break sectional SEM image of ANF nanopapers, d) ANF‐BS micropapers, and e) ANF‐BSNs composite nanopapers. f) EDX image of ANF‐BSNs composite nanopapers with 1.0 wt% BSNs and its EDX elemental mappings of Si in the tensile break sectional surface. g) The SEM image of ANF‐BS micropapers and h) ANF‐BSNs composite nanopapers (1.0 wt%). i) The schematic illustration of stress conduction in ANF nanopapers and ANF‐BSNs composite nanopapers.

Here, inspired by natural nacre, the whole 3D interconnective ANF in the ANF‐BSNs composite nanopapers is considered a framework that hosts numerous BSNs. Additionally, the presence of ANF in composite nanopapers enhances stress transfer between the layered BSNs reinforcements. In order to investigate the role of BSNs on the mechanical properties of the ANF‐BSNs composite nanopapers, the tensile strength and tensile modulus of ANF nanopapers, ANF‐BS micropapers, and ANF‐BSNs composite nanopapers were measured (**Figure** **5**a,b). The tensile strength, tensile modulus, and toughness of ANF are 143.87 MPa, 1.44 GPa, and 11.74 MJ m^−3^, respectively. After adding BS to ANF, the prepared ANF‐BS micropapers exhibit the lowest tensile strength and tensile modulus. This is because a loose structure and many defects are formed in ANF‐BS micropapers due to the weak interfacial interaction between ANF and BS (Figure 5c,d). With increasing BSNs content, the tensile strength and modulus of ANF‐BSNs composite nanopapers initially increase and then decrease, reaching their maximum values at a BSNs content of 1.0 wt%. At this BSNs content, the ANF‐BSNs composite nanopapers demonstrate a maximal tensile strength of 269.40 MPa, a modulus of 4.08 GPa, and a toughness of 16.22 MJ m^−3^. These properties demonstrate an increase of 87%, 183%, and 38%, respectively, compared to those of the control ANF nanopapers. Impressively, the ANF‐BSNs composite nanopapers with 1.0 wt% BSNs also exhibit excellent mechanical properties compared to other ANF‐based composite papers or films (Table S6, Supporting Information), which is confirmed by the performance in practical application (Movie S1, Supporting Information). Meanwhile, it can be observed that the dense layered structure is gradually formed in the composite nanopapers as BSNs mass fraction increases, and the clearest layered structure is observed in the ANF‐BSNs composite nanopapers with 1.0 wt% BSNs (Figure 5e). Additionally, the ANF‐BSNs composite nanopapers containing more than 1.0 wt% BSNs exhibit a disordered structure (Figure S9, Supporting Information), leading to a decrease in their tensile strength, modulus, and toughness. This phenomenon could be because excessive BSNs weaken interfacial interaction.^[^ ^46^, ^47^ ^]^ This disordered structure would also diminish their ability to block water molecules from entering the nanopapers (Figure 4c). Moreover, the corresponding energy‐dispersive X‐ray (EDX) element mapping images confirm the uniform distribution of BSNs in the ANF‐BSNs composite nanopapers with 1.0 wt% BSNs (Figure 5f; Figure S10, Supporting Information).

The outstanding mechanical performances of ANF‐BSNs composite nanopapers can be attributed to the layered “brick‐and‐mortar” structure. Compared to traditional fillers such as BS, ultrathin BSNs exhibit excellent interfacial adhesion with ANF due to their large specific surface area and multifunctional groups (Figure 5g,h). The continuous interconnected 2D nanosheets in ANF‐BSNs composite nanopapers eliminate the ubiquitous adverse stress concentrations in ANF nanopapers, thereby enhancing the strength of the composite nanopapers. Besides, the addition of electronegative BSNs would reduce the agglomeration of ANF in ANF‐BSNs composite nanopapers due to the electrostatic repulsive force. Furthermore, the 3D architecture of ANF‐BSNs composite nanopapers maximized the interfacial contact area between the filler and matrix, leading to efficient load transfer^[^ ^11^ ^]^ (Figure 5i).

### 2.4 Electrical Characterization of ANF‐Based Composite Nanopapers

**Figure 6:**
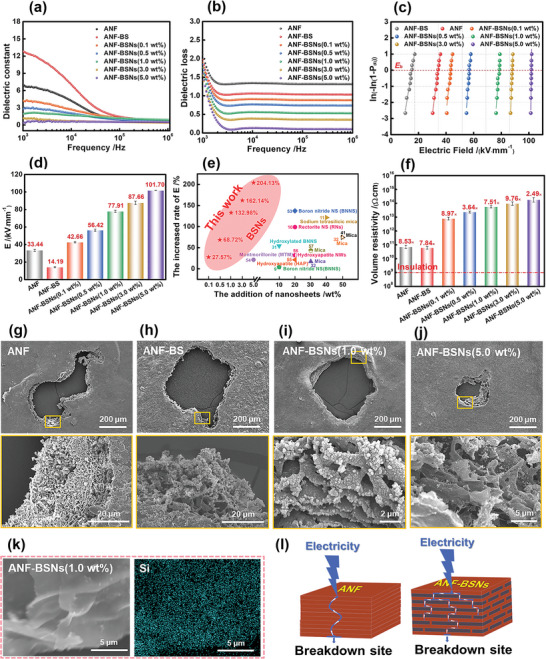
Electrical insulating performance of the ANF‐BSNs composite nanopapers. a) The dielectric constant, b) dielectric loss, c) failure probability of dielectric breakdown deduced from the Weibull distribution, and d) the dielectric breakdown strength of ANF nanopapers, ANF‐BS micropapers, and ANF‐BSNs composite nanopapers. e) Comparison of the increased rate of dielectric strength in different addition of as‐prepared ANF‐BSNs composite nanopapers and previously reported nanosheets‐based composites; f) Volume resistivity of ANF nanopapers, ANF‐BS micropapers, and ANF‐BSNs composite nanopapers; g) SEM images of dielectric breakdown sites of ANF nanopapers, h) ANF‐BS micropapers, i) ANF‐BSNs composite nanopapers with 1.0 wt% BSNs, and j) ANF‐BSNs composite nanopapers with 5.0 wt% BSNs. k) EDX image of dielectric breakdown site in ANF‐BSNs composite nanopapers with 1.0 wt% BSNs and their EDX elemental mapping of Si in the dielectric breakdown site surface. m) The schematic models of electric current transfer in ANF nanopapers and ANF‐BSNs composite nanopapers.

In order to investigate the capacity of composite micropapers and nanopapers in storing electrical energy under an electric field, the dielectric constant and loss factor were measured ranging from 10^3^ to 10^6^ Hz at room temperature by using an Agilent 4284A precision LenZ‐Capacitor‐Resistance (LCR) meter. The dielectric constant of the ANF‐BSNs composite nanopapers exhibits a lower dielectric constant than the control ANF nanopapers. This is primarily ascribed to the presence of BSNs with abundant functional groups. These BSNs enhance the dispersion ability of ANF, preventing its agglomeration. Therefore, BSNs can form chemical bonds with dispersed ANF through hydroxyl interaction, resulting in a decrease in the number of free polar groups in ANF‐BSNs composite nanopapers. These polar groups exhibit enhanced mobility in a changing electric field and a decreasing polarization effect that would affect the dielectric constant.^[^ ^48^, ^49^, ^50^ ^]^ In addition, the dielectric constant of ANF‐BSNs composite nanopapers continues to decrease as BSNs content increases, indicating that the storage and transfer of electrical energy in an electric field are suppressed due to the addition of the BSNs in the composite nanopapers (**Figure** **6**a).

The dielectric loss represents the energy dissipation in the process of releasing heat from a dielectric under an Alternating Current electric field. As shown in Figure 6b, the dielectric loss of ANF‐BSNs composite nanopapers remains at a low value in the range of 0.13–0.91. Simultaneously, their interfacial polarization decreases as BSNs content increases. Because of the decrease in polarization, their energy loss caused by thermal motion under the influence of an electric field force decrease, so there is a corresponding decrease in dielectric loss.^[^ ^49^, ^50^, ^51^ ^]^ Notably, the dielectric loss of ANF‐BSNs composite nanopapers is significantly decreased to 0.13 (at 10^4^ Hz) when the BSNs content is merely 5.0 wt%. The excellent property of low dielectric loss can be attributed to the dense and interconnected layered “brick‐and‐mortar” structure in ANF‐BSNs composite nanopapers and excellent interfacial adhesion between BSNs and ANF. These advantages in ANF‐BSNs composite nanopapers structure effectively prevent current leakage and restrict the movement of dipole molecules in the nanopapers, reducing friction and minimizing heat loss caused by their repetitive arrangement.^[^ ^52^ ^]^ Therefore, due to their outstanding dielectric performance, ANF‐BSNs composite nanopapers are expected to be utilized as high‐performance insulating materials.

The dielectric breakdown strength (*E*_b_) represents the capacity of the insulating material to withstand voltage, which directly reflects the electrical properties of advanced electronics and power equipment. In order to analyze the *E*_b_ of these composite films, the two‐parameter Weibull distribution function is applied to fit their failure statistics (Figure 6c). The *E*_b_ of ANF nanopapers is only 33.44 kV mm^−1^, and it can be observed from Figure 6m that the carbonization occurs on the surface of the dielectric breakdown site due to the local high temperature caused by the strong current. While ANF‐BS micropapers exhibit the lowest *E*_b_ of 14.19 kV mm^−1^, mainly because the lamellar structure becomes loose and a few cavitations appear.^[^ ^32^ ^]^ The ANF‐BSNs composite nanopapers with 5.0 wt% BSNs exhibit the highest *E*_b_ of 101.70 kV mm^−1^, which is 204% higher than that of the control ANF nanopapers (Figure 6d). Meanwhile, the increased *β* value, which represents the shape parameter associated with the width of the distribution of the Weibull modulus, indicates the excellent reliability of the ANF‐BSNs composite nanopapers. Currently, numerous composite films or papers loaded with inorganic nanosheets have demonstrated remarkable dielectric breakdown strength. Their breakdown strength exhibits a significantly increased rate (up to 138%) when adding different nanosheets between 5 and 50 wt% compared to the parallel sample.^[^ ^9^, ^10^, ^11^, ^31^, ^32^, ^41^, ^53^, ^54^, ^55^, ^56^, ^57^ ^]^ Nevertheless, in this work, the ANF‐BSNs composite nanopapers containing only 1.0 wt% BSNs exhibit an impressive increase of 133% in breakdown strength. These results confirm that the increasing rate of *E*_b_ after the addition of BSNs is higher than that of other nanosheets (Figure 6e; Table S7, Supporting Information).

Compared to the control ANF nanopapers, the relatively smaller dielectric breakdown sites of ANF‐BSNs composite nanopapers verified the contribution of the microstructural design in ANF‐BSNs composite nanopapers (Figure 6g–j; Figure S11, Supporting Information). Taking the ANF‐BSNs composite nanopapers with 1.0 wt% BSNs as an example, ANF and flake‐like BSNs form a highly ordered lamellar and densely packed structure along the in‐plane direction, resulting in reducing the intensity of current spread per unit area.^[^ ^58^, ^59^ ^]^ Besides, the corresponding EDX element mapping images verify the uniform distribution and lamellated structure of BSNs in the ANF‐BSNs composite nanopapers with 1.0 wt% BSNs (Figure 6k; Figure S12, Supporting Information). The mechanism by which BSNs enhanced the electrical insulating performances of ANF‐BSNs composite nanopapers can be explained from the schematic models (Figure 6m). Compared to the control ANF nanopapers, ANF‐BSNs composite nanopapers exhibit a consolidated nacre‐mimetic structure with fewer micro‐scaled voids, which facilitate lateral dissipation of injected charges and provide tortuous paths for the propagation of electric trees, as well as reducing the possibility of charge injection. In addition, under the applied electric field, electrons tend to be attracted toward BSNs with high dielectric strength, which makes the internal charge migrate toward BSNs.^[^ ^11^ ^]^ Therefore, the breakdown mechanism of ANF‐BSNs composite nanopapers with a nacre‐mimetic structure can be summarized as the avalanche‐type electronic breakdown mechanism rather than the electrothermal breakdown of ANF nanopapers (Figure 6g). Moreover, ANF‐BSNs composite nanopapers also exhibit superior dielectric strength in a 150 °C silicone oil bath, which verifies their excellent dielectric performance in practical insulating applications (Figure S13, Supporting Information).

Additionally, the volume resistivity is further analyzed and displayed in Figure 6f. The introduced BSNs demonstrate a significantly positive effect on improving the volume resistivity compared to the control ANF nanopapers (8.53 × 10^10^ Ω cm). Typically, the volume resistivity of ANF‐BSNs composite nanopapers containing only 1.0 wt% BSNs reaches a remarkable value of 7.51 × 10^13^ Ω cm, indicating an impressive enhancement of 879 times. This can be primarily attributed to the densely layered “brick‐and‐mortar” structure formed in the internal ANF‐BSNs composite nanopapers, which decreases its internal free volume, thus causing a decrease in carrier mobility.^[^ ^60^, ^61^ ^]^ In fact, their volume resistivity surpasses the evaluation of electrical insulation (10^9^ Ω cm), thereby contributing to achieving better safety.

## 3 Conclusion

In summary, ultrathin BSNs with high aspect ratios (≈780.1) were successfully fabricated from BS through a chemical/mechanical approach. The obtained BSNs were homogeneously dispersed in an ethanol solution and contained abundant hydroxyl groups on their surface, which were beneficial for various applications. Inspired by natural nacre, the BSNs serving as ideal building blocks were co‐assembled with ANF into a novel nacre‐mimetic ANF‐BSNs composite nanopapers through a vacuum‐assisted filtration method. Impressively, the intrinsic merits of BSNs and ANF as well as the nacre‐mimetic microstructure impart ANF‐BSNs composite nanopapers with exceptional mechanical properties even when containing an ultralow BSNs content of 1.0 wt%. Specifically, the tensile strength, modulus, and toughness are 269.40 MPa, 4.08 GPa, and 16.22 MJ m^−3^, respectively, which are 87%, 183%, and 38% higher compared to the control ANF nanopapers. Meanwhile, their electrical dielectric breakdown strength reaches 77.91 kV mm^−1^, an increase of 133% in comparison with the control paper. These superiorities are wonderfully integrated with outstanding bendability, remarkable volume resistivity, and extremely low dielectric constant and loss, which are far superior to conventional acre‐mimetic composite nanopapers. This work provides an efficient approach for producing high‐quality BSNs and demonstrates a promising strategy for developing advanced electrical insulating composites for modern miniaturized, high‐power electrical equipment.

## 4 Experimental Section

### Materials

Basalt scales were kindly provided by Hongfa Basalt material Co., Ltd. (Dandong, China). Aramid fibers (Twaron) were obtained from Du Pont Co., Ltd. HCl, anhydrous ethanol, sodium chloride (NaCl), Sodium hydroxide (NaOH), ammonium chloride (NH_4_Cl), DMF, dimethyl sulfoxide (DMSO), and phenolphthalein were all purchased from Sinopharm Group Chemical Reagent Co., Ltd. (Shanghai, China). Lithium chloride (LiCl), PVP, and potassium hydroxide (KOH) were all purchased from Macklin Biochemical Co., Ltd. (Shanghai, China). DIW was employed in all experiments. All chemicals and solvents used in the experiment were of guaranteed analytical grade and without purification.

### Preparation of BSNs

In the typical experiment, 0.4 g of basalt scales (oven dried) were chemically etched using 4.0 m HCl with continuous stirring at 80 °C for 12 h, aiming to remove the metal oxide on the surface of BS and obtain a rough surface. The obtained suspension was repeatedly washed with DIW until pH reached ≈7 in order to remove the dissolved metal ions and residual HCl. The resulting sample was named HCl‐BS. Subsequently, the aforementioned dried sample was mixed with 200 mL of saturated NaCl solution and stirred at 110 °C for 8 h to remove impurity ions. The resulting sample was repeatedly washed with anhydrous ethanol and DIW until the complete removal of Cl^−^. Next, this sample was mixed with 200 mL of LiCl solution (3.0 mol L^−1^) in a 500 mL flask and stirred at 110 °C for 8 h. The obtained suspension was repeatedly washed with anhydrous ethanol and DIW until the removal of Li^+^. The resulting sample was named Li^+^‐BS. Then, Li^+^‐BS with a concentration of 1.0 wt% was treated in the ultrasonic cell grinder at 800 W for 1 h. After ultrasonication, the suspension was left undisturbed at 25 °C for 24 h, and then the turbid mixture was centrifuged at 500 rpm (57.95 g) for 30 min, and the supernatant was collected. The resulting dispersion was named BSNs. To determine the concentration of BSNs in the supernatant, 30 mL of solution was taken out of the supernatant and filtered through a porous nylon membrane. Then the wet membrane was weighed after drying in a vacuum oven at 100 °C for 12 h. The concentration of BSNs in BSNs supernatant is calculated as follows:

(1) $$C_{BSNs}=\frac{m_{2}-m_{1}}{v}$$

where *C*_BSNs_ represents the concentration of BSNs (µg mL^−1^), *m*_1_ is the mass of the control nylon membrane after drying in a vacuum oven at 100 °C for 12 h (µg), *m*_2_ is the mass of the membrane after BSNs are filtered through and then dried in a vacuum oven at 100 °C for 12 h (µg). *v* is the volume of the BSNs taken out of the supernatant and then filtered through a porous nylon membrane (mL).

In addition, the yield and production rate of BSNs can be calculated as follows:

(2) $$Y_{BSNs}=v\times C_{BSNs}\times 10^{-3}$$

(3) $$P_{BSNs}=\frac{v\times C_{\mathrm{BSNs}}}{t}\times 10^{-3}$$

where *Y*_BSNs_ and *P*_BSNs_ are the yield (mg) and production rate (mg h^−1^) of BSNs, respectively; *C*_BSNs_ is the concentration of BSNs (µg mL^−1^), *v* is the volume of the produced BSNs (mL), and *t* is the time for producing the above BSNs (h).

### Preparation of ANF

A stable and uniform ANF solution was synthesized from PPTA fibers, according to the previous report.^[^ ^40^ ^]^ Briefly, 1.0 g of aramid fibers were initially immersed in KOH solution (1.5 g of KOH mixed with 20 mL of DIW) at 25 °C. Next, this sample was mechanically stirred in DMSO solution (500 mL, 99.9%) at room temperature, and the mixed system experienced magnetic stirring at 25 °C for 1 h under a sealed environment. Based on the specific volume ratio of 2:1, 200 mL of DIW was further injected into 100 mL of ANF solution while maintaining constant mechanical stirring. Subsequently, anhydrous ethanol and deionized water were employed alternately to remove the excess DMSO and KOH present in the ANF solution, assisted by vacuum filtration to acquire purified colloidal ANF. DIW was added to the purified colloidal ANF until the total volume reached 400 mL, resulting in an ANF suspension with a concentration of 0.4 mg mL^−1^. The ANF suspension was then stored at 4 °C for further use.

### Preparation of ANF‐BSNs Composite Nanopapers

First, different volumes of BSNs suspension were dispersed into the obtained ANF dispersion under high‐speed shearing treatment to obtain homogeneous ANF‐BSNs dispersions with different BSNs contents. Then, the mixed solution was further stirred for 30 min, and the ANF‐BSNs wet sheets were formed by vacuum filtration. Next, the formed wet sheets were dried at 105 °C for 10 min, leading to the formation of ANF‐BSNs composite nanopapers. ANF nanopapers and ANF‐BS micropapers were prepared using the same method as described above.

### Sample Characterization

Zeta potential of BS, HCl‐BS, Li^+^‐BS, and BSNs was measured by a Zeta potential analyzer (Nano ZS‐90, Malvern, England). The chemical functional groups of BS, HCl‐BS, Li^+^‐BS, and BSNs were characterized by FT‐IR (Vertex70, Bruker, Germany) and Raman spectroscopy (THEM, USA). The FTIR and Raman characterizations were performed under a scanning range of 500–4000 cm^−1^ and 532 nm excitation, respectively. XRD data were collected using X‐ray diffractometer (D8 Advance, Bruker, Germany) with Cu Kα (λ = 0.1542 nm). The patterns were recorded from 2θ = 5° to 2θ = 80° at 40 V and 40 mA. The step size and scanning speed were 0.2° and 6° min^−1^, respectively. The elemental composition and chemical state were analyzed using XPS instruments (Thermo Fisher Scientific, ESCALAB Xi^+^, England). The morphological features were characterized using SEM (JEM 2100, JEOL, Japan). The chemical composition was measured by EDX. Surface roughness and thickness were investigated by AFM (Dimension Icon, Bruker Nano, USA). The morphology of BS, Li^+^‐BS, BSNs, and ANF was observed by TEM (Tecnai G2 F20 S‐TWIN, FEI, USA) at 200 kV. The tensile strength of the as‐prepared micropapers and nanopapers was measured by a tensile testing instrument (AI‐7000‐NGD, Goodtechwill, China) equipped with a 500 N load cell at a load speed of 5 mm min^−1^ with a gauge length of 20 mm. The size of the samples was 10 mm × 50 mm. The reported average values were calculated as averages of six test results for each sample. The thickness of samples was measured in accordance with TAPPI Standard Method T411 (T411 om‐89). For data accuracy, five specimens were used for testing. The thermal stability properties of composite nanopapers were also monitored by thermogravimetric analysis (TGA, Q5000IR, TA) under a nitrogen atmosphere from 25 to 800 °C with a heat ramp rate of 10 °C min^−1^. The loading content of BSNs in ANF‐BSNs composite nanopapers is calculated as follows:

(4) $$C_{ANF}+C_{BSNs}=1$$

(5) $$0.3953C_{ANF}+0.9302C_{BSNs}=0.4003$$

where *C*_ANF_ and *C*_BSNs_ are the mass ratios of ANF and BSNs in ANF‐BSNs composite nanopapers, respectively. 0.3953, 0.9302, and 0.4003 are the mass residual of ANF, BSNs, and ANF‐BSNs composite nanopapers at 800 °C in thermos degradation curves, respectively.

### Cation Exchange Capacity Testing

CEC was utilized to evaluate the capability of the material to exchange the inside cations (e.g., K^+^, Na^+^, Ca^2+^, Mg^2+^, Al^3+^, etc.) under neutral conditions. In the typical experiment, ≈3 g of sample (oven dried) was washed three times using 25 mL of ethanol solution (70%). Then, 25 mL of the extracted solution containing ammonium chloride and ethanol solution was added to the washed sample with continuous stirring at 25 °C for 30 min. Next, the mixture was left undisturbed for 8 h to obtain the upper liquid. This extraction process was repeated three times to obtain almost 75 mL of reacted extracted solution. Subsequently, 25 mL of the reacted extracted solution was heated to a boiling point, 8 mL of formaldehyde solution and 5 drops of phenolphthalein indicator (0.1%) were added, and 0.1 mol L^−1^ sodium hydroxide solution was employed to titrate the mixture solution until its color changed to pink. CEC can be calculated using the following equation:

(6) $$CEC=\frac{c_{1}\times \left(v_{2}-v_{1}\right)\times 100}{m\times 25}$$

where CEC is the cation exchange capacity (mmol g^−1^), c_1_ is the concentration of sodium hydroxide (mol L^−1^), v_1_ and vV_2_ represent the dosage of sodium hydroxide in titrating the reacted and unreacted extracted solution (mL), respectively, and *m* is the mass (g) of the samples.

### Electrical Properties Testing

The dielectric constant and loss factor of the ANF nanopapers, ANF‐BS micropapers, and ANF‐BSNs composite nanopapers were measured using an LCR meter (Agilent Tech., E4990A, USA) within a frequency range (from 1 kHz to 1 MHz). All samples were coated with a conductive layer and measured at room temperature. The relatively high tolerance of the reported dielectric constants arises from the uncertainty of the measured electrode separation distance because a marginal diffusion of copper into the nanocomposites is expected during the preparation of electrodes via sputtering.^[^ ^9^ ^]^ The volume resistivity of ANF nanopapers, ANF‐BS micropapers, and ANF‐BSNs composite nanopapers was characterized by an Agilent 4339B high resistance meter (Ω cm) (BEST‐212, Beiguangjing Instrument Equipment Co., Ltd., China). The DC breakdown strength of the prepared micropapers and nanopapers was carried out on a voltage‐withstand tester (CS2672D, Nanjin Changsheng Co., Ltd., China) at room temperature with ≈50% relative humidity. Hence, the Weibull distribution function is obtained by the following equation:

(7) $$P_{\left(E\right)}=1-\exp\left[-{\left(E/E_{B}\right)}^{\beta }\right]$$

where *P*_(E)_ represents the cumulative probability of electrical failure, and *E* is the experimental breakdown strength of these composite micro‐papers and nanopapers. *E*_b_ is the characteristic breakdown electric field strength at which 63.2% of the samples have failed. *β* represents the shape parameter associated with the width of the distribution of the Weibull modulus.

## Conflict of Interest

The authors declare no conflict of interest.

## Supporting information

Supporting Information

Supplemental Movie 1

## Data Availability

The data that support the findings of this study are available from the corresponding author upon reasonable request.
