# Supplementary material for: Novel Fabrication of Basalt Nanosheets with Ultrahigh Aspect Ratios Toward Enhanced Mechanical and Dielectric Properties of Aramid Nanofiber‐Based Composite Nanopapers
Source: Adv Sci (Weinh). 2023 Jul 23;10(27):2302371. doi: 10.1002/advs.202302371 (PMC10520689; doi:10.1002/advs.202302371)
Supplement: Supplementary file 1 — Supporting Information [file ADVS-10-2302371-s002.pdf]

ADVANCED SCIENCE
Open Access

Supporting Information

for *Adv. Sci.*, DOI 10.1002/advs.202302371

Novel Fabrication of Basalt Nanosheets with Ultrahigh Aspect Ratios Toward Enhanced Mechanical and Dielectric Properties of Aramid Nanofiber-Based Composite Nanopapers

Dexian Ji, Shunxi Song, Yuming Lyu, Wei Ren, Linghao Li, Bin Yang and Meiyun Zhang**

Supporting Information

Novel Fabrication of Basalt Nanosheets with Ultrahigh Aspect Ratios toward Enhanced Mechanical and Dielectric Properties of Aramid Nanofiber-Based Composite Nanopapers

Dexian Ji, Shunxi Song, Yuming Lyu, Wei Ren, Linghao Li, Bin Yang, Meiyun Zhang**

D. Ji, S. Song, Y. Lyu, W. Ren, L. Li, B. Yang, M. Zhang

Key Laboratory of Auxiliary Chemistry and Technology for Chemical Industry, Ministry of Education

Shaanxi Province Key Laboratory of papermaking Technology and Specialty paper Development

College of Bioresources Chemical and Materials Engineering

Shaanxi University of Science & Technology

Xi’an 710021, P. R. China

E-mail: songshunxi@sust.edu.cn; myzhang@sust.edu.cn

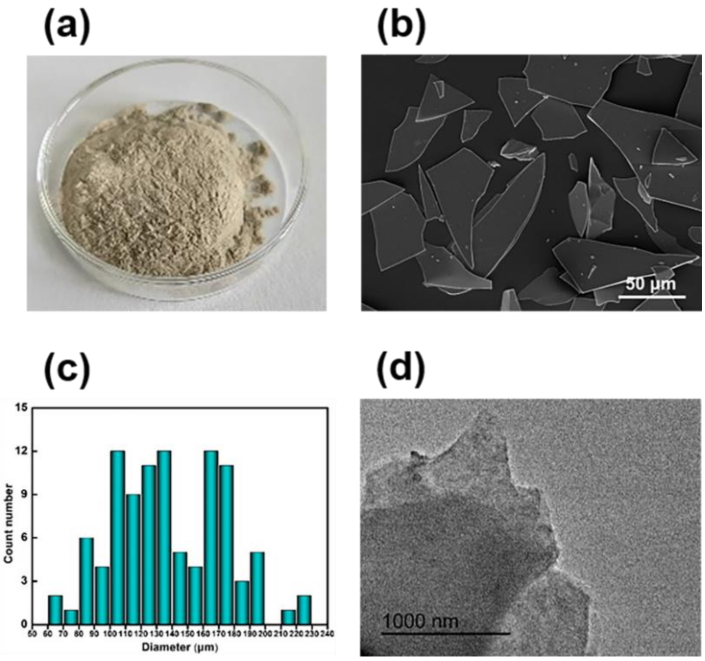

Figure S1. Characterization of basalt scales (BS). a) Photograph and (b) scanning electron microscopy (SEM) image of BS. c) Statistics of lateral size of BS. d) Transmission electron microscopy (TEM) image of BS.

Table S1 XFR analysis of BS, HCl-BS, Li^+-BS, and BSNs.

**Elements**	**Sample /%**			
	BS	HCl-BS	Li^+-BS	BSNs
**Si**	41.15	52.67	62.98	46.99
**Al**	16.05	14.33	9.49	15.38
**Fe**	11.46	7.84	5.42	8.42
**Na**	10.10	7.97	2.81	5.16
**Ca**	9.69	7.44	4.64	10.54
**Mg**	5.48	4.91	3.17	8.35
**Li**	/	/	11.49	5.16

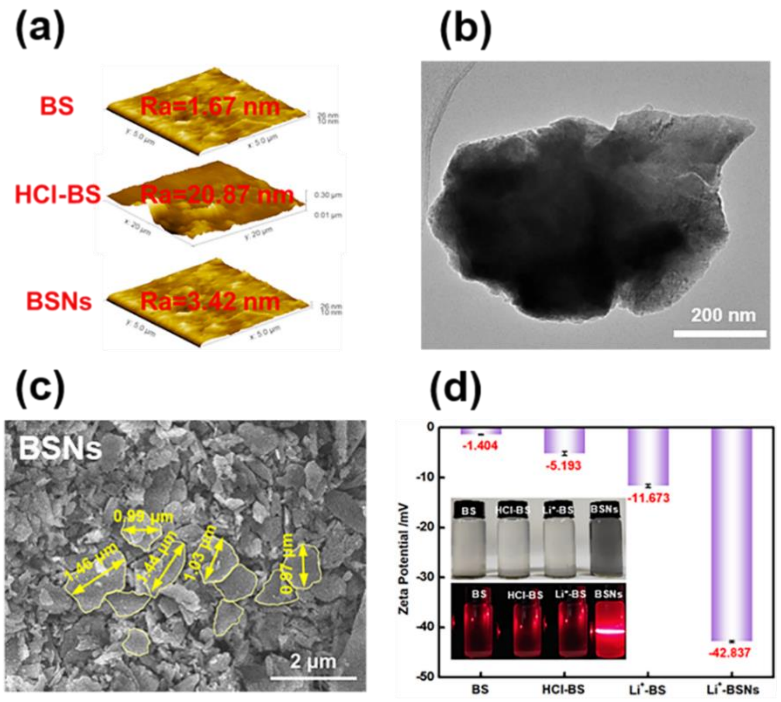

Figure S2. a) The surface roughness (Ra) of BS, HCl-BS, and BSNs; b) The TEM image of BSNs. The dark area may be relative to that of the Fe^{2+} in BSNs which can affect the TEM test[1]. c) SEM micrographs of BSNs. d) Zeta potential, digital photos of BS, HCl-BS, Li^{+}-BS, and BSNs.

Table S2 The cation exchange capacity of BS, HCl-BS, and Li^{+}-BS

**Sample**	**m_s**	**V_2**	**V_1**	**C(NaOH)**	**CEC(mmol·g^{-1})**	
	3.0098		30.70		9.60	
**BS**	3.0152		30.90		7.32	**9.64**
	2.9752		30.50		12.0	
	2.9966		30.10		16.45	
**HCl-BS**	2.9742	31.55	29.90	0.0085	18.86	**16.46**
	3.0219		30.30		14.06	
	2.9076		29.90		19.29	
**Li^{+}-BS**	2.9045		29.60		22.83	**22.79**
	2.9142		29.30		22.79	

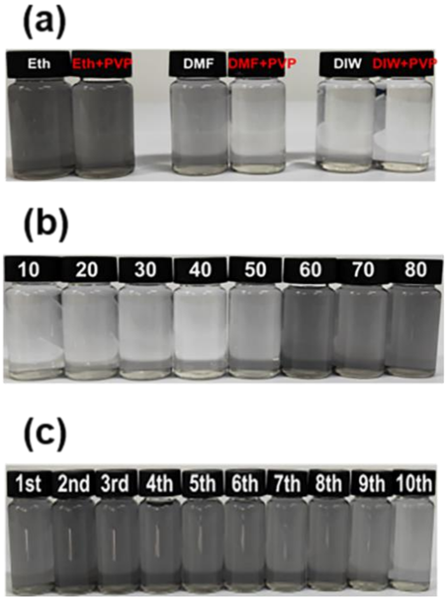

Figure S3. a) Photograph of the BSNs dispersions prepared by 1 h ultrasonication (800 W) of Li^+-BS (10 mg·mL^{-1}) with different solvents (mechanical stirring for 8 h) and PVP (1.0×10^{-3} M); b) Photograph of BSNs dispersions obtained by different ultrasonic time; c) Photograph of BSNs dispersions obtained by different cycle times of Li^+-BS (10 mg·mL^{-1}) and ethanol mixture.

Table S3 The concentration and yield of BSNs prepared by Li^+-BS in different solvents.

**Dispersion solvent**	**Concentration (μg·mL^{-1})**	**Production rate (mg·h^{-1})**	**Yield (mg)**
Ethyl alcohol	35.2	7.0	7.0
Ethyl alcohol+PVP	27.1	5.4	5.4
DMF	25.2	5.0	5.0
DMF+PVP	20.9	4.2	4.2
DIW	17.1	3.4	3.4
DIW+PVP	16.0	3.2	3.2

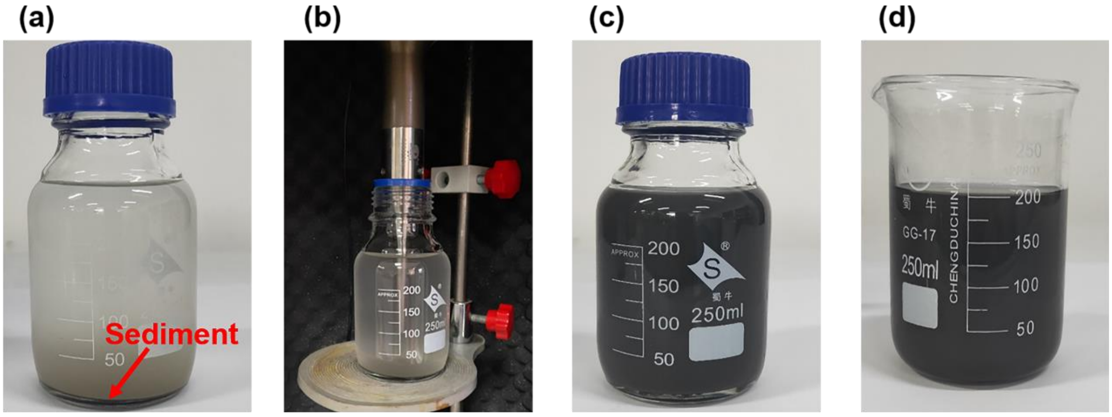

Figure S4. a) Photographs of the mixture containing Li^{+}-BS and ethanol (Li^{+}-BS with a concentration of 1.0 wt%). The large scale and weight induce the rapid sedimentation (almost 10 min) of the Li^{+}-BS; b) Photograph of a 15 mm ultrasonic horn with ultrasonication power of 800 W in 250 mL of Li^{+}-BS dispersion (Li^{+}-BS with a concentration of 1.0 wt%); c) Photograph of the BSNs dispersed in 250 mL ethanol after the ultrasonication for 1 h; d) Photograph of BSNs obtained from the top 4/5 of the supernatant which was stood at 25 °C for 24 h and then centrifuged at 500 rpm for 30 min.

Table S4 The concentration and yield of BSNs prepared by Li^{+}-BS in different ultrasonic time

**Ultrasonic time (min)**	**Concentration ($\mu g \cdot mL^{-1}$)**	**Production rate ($mg \cdot h^{-1}$)**	**Yield (mg)**
10	5.2	6.2	6.2
20	9.1	5.5	5.5
30	14.1	5.6	5.6
40	20.8	6.2	6.2
50	31.2	7.5	7.5
60	35.2	7.0	7.0
70	36.8	6.3	6.3
80	38	5.7	5.7

Table S5 The concentrations and yield of BSNs dispersions obtained by different cycle times of Li^+-BS and ethanol mixture.

Exfoliation cycles	Concentration ($\mu g\cdot mL^{-1}$)	Production rate ($mg\cdot h^{-1}$)	Yield (mg)
1st	35.2	7.0	7.0
2nd	45.3	9.1	9.1
3rd	41.3	8.3	8.3
4th	40.1	8.0	8.0
5th	36.3	7.3	7.3
6th	34.7	6.9	6.9
7th	33.3	6.7	6.7
8th	30.7	6.1	6.1
9th	28.3	5.7	5.7
10th	28.3	5.7	5.7

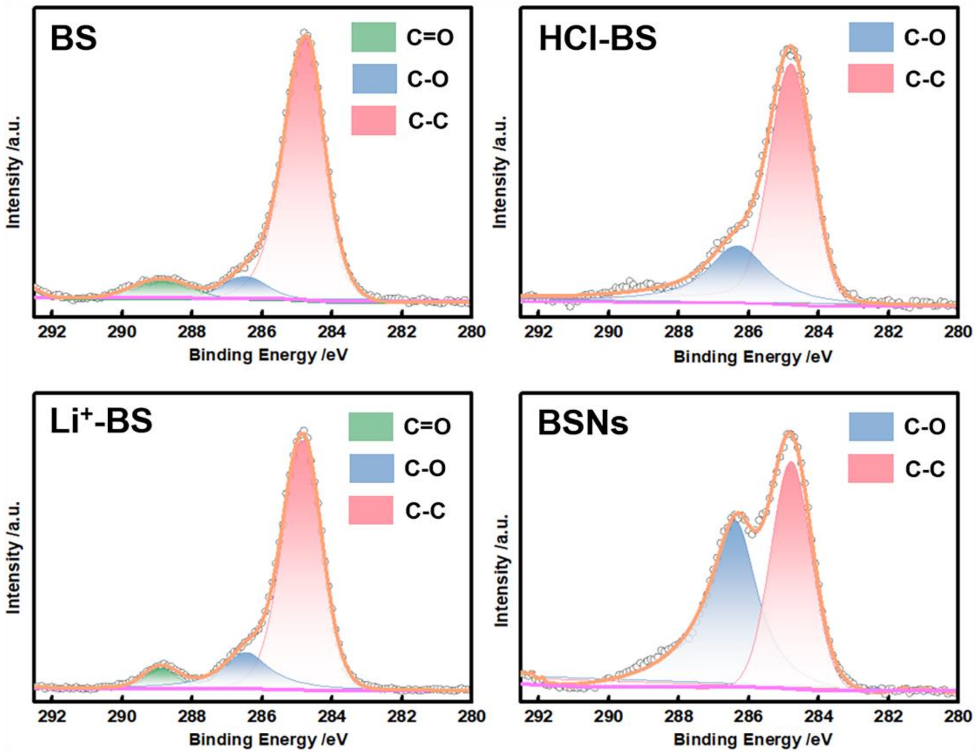

Figure S5. XPS C 1s of BS, HCl-BS, Li^+-BS, and BSNs.

As shown in **Figure S5**, the fitted peak situated at 284.8 eV represents C-C groups, and the two other peaks located at 286.2 eV and 289.0 eV belong to C-O and C=O groups[2], respectively. This is probably related to the adsorption of CO_2 and exposure of the internal oxides such as CaO and MgO in the above samples.

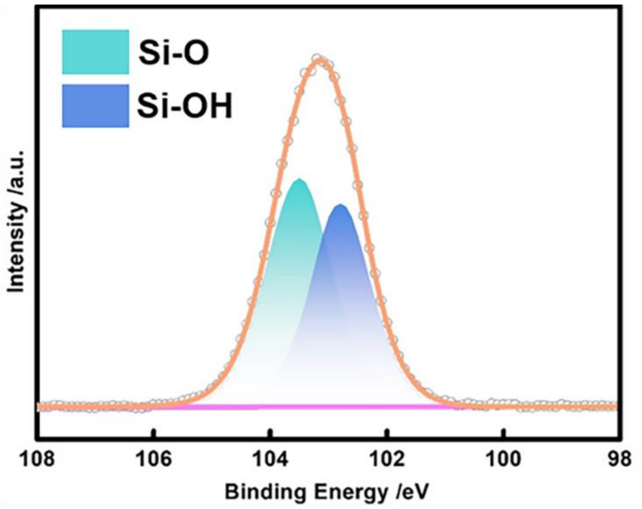

Figure S6. XPS Si 2p of Li^+-BS.

No characteristic peak of Li 1s was found in Li^+-BS **(Figure S6)**, probably because the injection of argon ion easily occurs on the surface of the testing material during the test process, resulting in the accumulation of positive charges on the sample surface. And the repulsion of the same charge will lead to the migration of positively charged Li^+ to the interior of testing materials, resulting in decreasing the content of Li elements on the material surface, which cannot be effectively detected by the detector[3].

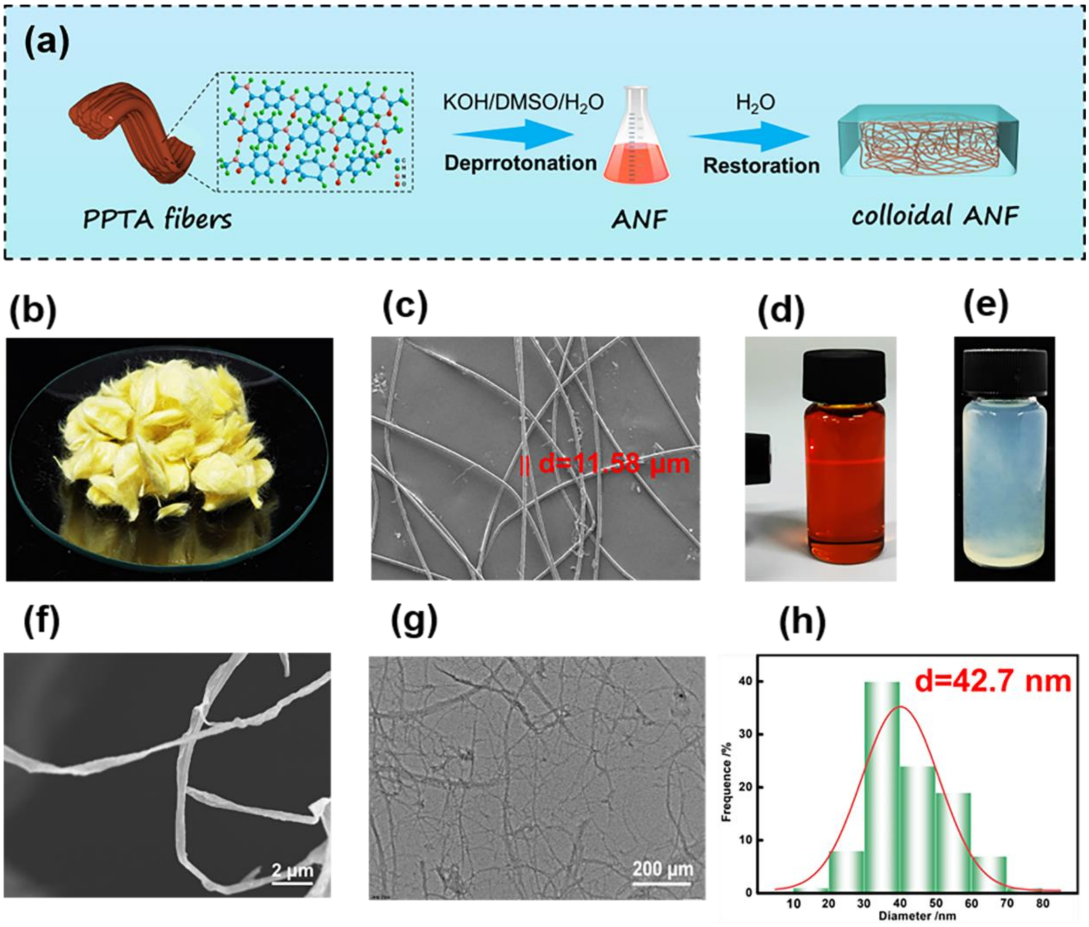

Figure S7. a) Schematic illustration of the fabrication process of ANF; Photograph (b), SEM image (c) of PPTA fiber; Photograph ANF/DMSO solution (d) ANF (e); SEM image (f), TEM images (g) and statistical analysis of diameter distributions (h) of ANF.

Since Takayanagi[4] first proposed the concept of deprotonation of PPTA fibers in NaOH/DMSO to obtain aramid nanofibers (ANF) in the early 1980s, the study of the preparation and application of ANF has been favored by a huge amount of researchers, and the excellent properties of ANF in mechanical, thermostability, flame retardancy, electrical insulation were gradually found out[5-7]. Alternatively, Yang[5] initially proposed the fabrication of ANF by deprotonating macroscopic PPTA fibers in a KOH/DMSO/H_2O system in 2019, shortening the preparation period of ANF from 7 days to 4 h. As illustrated in **Figure S7a,** proton donor-assisted deprotonation was proposed for the preparation of ANF.

PPTA fibers, the precursor of ANF, is para-linked by rigid macromolecular chains, which is attributed to strong atomic bonds along the axial orientation and

weak interchain bonds along the radial orientation this arrangement endows the fibers with anisotropic characteristics and impressive strength[5] (**Figure S7a-c**). The efficiency of the preparation of ANF is closely related to the concentration of -OH in the reaction system. To enhance the concentration of -OH, we modified the preparation system by using a KOH solution instead of solid KOH. Moreover, we proposed a donor-assisted deprotonation method, which significantly reduced the preparation time to just 1 hour. As shown in **Figure S7d,** the homogeneous transparent dark red ANF/DMSO solution was obtained in 1 h. Due to the lack of hydrogen from the amide groups, the molecular structure of ANF in DMSO solution is incomplete. Therefore, Proton donors (DI water), can be used to structurally restore the ANFs, achieving the nanofibers with an integrated molecular structure (**Figure S7e**). As displayed in **Figure S7f-h**, PPTA fiber was successfully exfoliated into nanoscaled fibers with an average diameter of 42.7 nm for only 1 h deprotonation reaction. The obtained ANF not only maintains the above-mentioned outstanding performance of macroscopic PPTA fiber but also exhibit nanoscale dimensions, a large aspect ratio, and high specific surface area, which could improve the poor interfacial bonding with other matrixes.

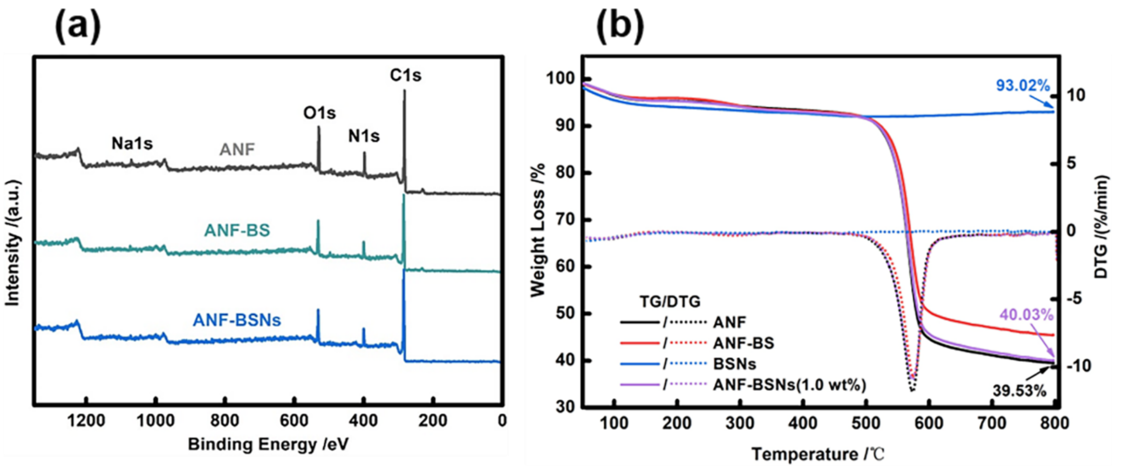

Figure S8. a) XPS spectra of ANF nanopapers, ANF-BS micropapers, and ANF-BSNs composite nanopapers; b) Thermogravimetric (TG) curves of ANF nanopapers, ANF-BS micropapers with 1.0wt% BS (denoted as ANF-BS) and ANF-BSNs composite nanopapers with 1.0wt% BSNs (denoted as ANF-BSNs).

Table S6 The summarized tensile strength parameters of the ANF-based composites

**Nanosheets**	**Device structure**	**Addition of Nanosheets**	**Tensile strength/ Mpa**	**Year** [Ref]
NTs	NTs/ANF nanopaper	40 wt%	175	2019[8]
BNNs	PANF-BNNS nanocomposites	10 wt%	120	2019[9]
MTM	ANF/MTM nanocomposite films	5 wt%	126.5	2020[10]
MWCNTs	MWCNTs/ANFs papers	20 wt%	82.1	2021[11]
RNs	ANF-RNs nanopapers	20 wt%	232	2022[12]
HAP	HAP/ANF nanocomposite papers	20 wt%	73.5	2022[13]
BNNS	BNNS/ANF films	30 wt%	100.8	2022[14]
BNNS	ANF/PVA/BNNS-OH composite film	5.0 wt%	329.6	2022[15]
Mica	ANF-PEI-Mica nanopapers	40 wt%	261	2023[16]
BSNs	ANF-BSNs nanopapers	0.1 wt%	149.3	**This work**
		0.5 wt%	197.4	
		1.0 wt%	269.4	
		3.0 wt%	153.5	
		5.0 wt%	65.9	

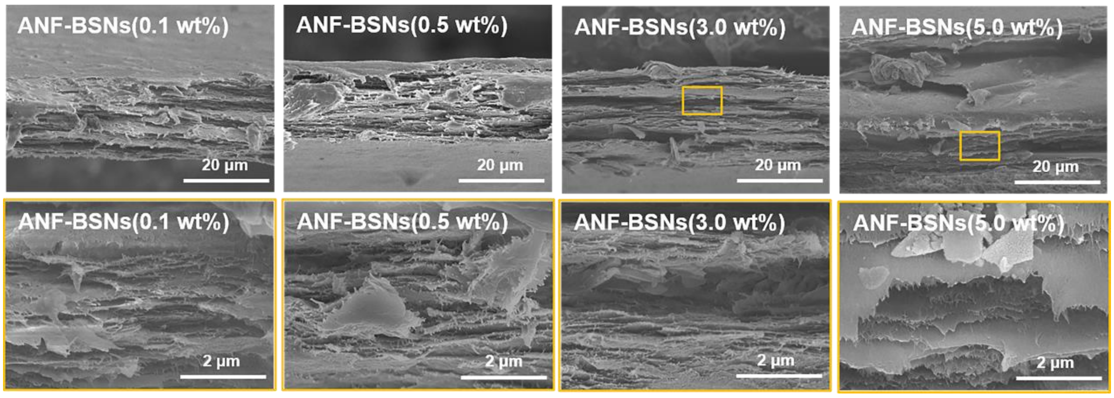

Figure S9. The tensile break sectional SEM image of ANF-BSNs composite nanopapers with different BSNs contents (0.1 wt%, 0.5 wt%, 3.0 wt%, and 5.0 wt%).

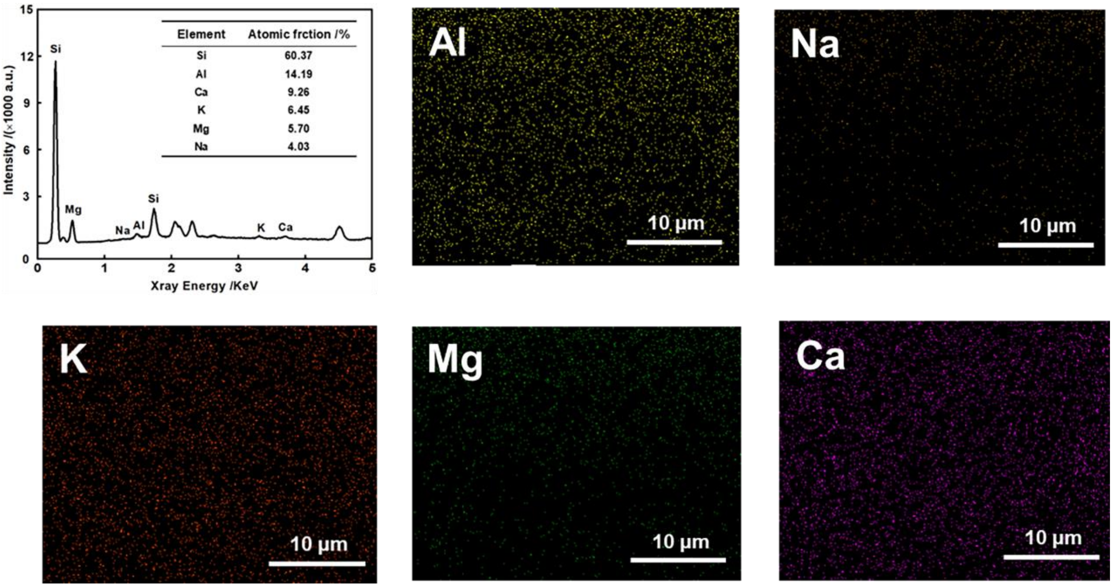

Figure S10. Energy-dispersive X-ray (EDX) image of ANF-BSNs composite nanopapers with 1.0 wt% BSNs.and its EDX elemental mappings of Al, Na, K, Mg, and Ca in the cross-sectional surface.

Table S7 The summarized dielectric breakdown strength parameters of composite films or papers loaded with inorganic nanosheets in recent years.

**Nanosheets**	**Device structure**	**Addition of Nanosheets**	**The increasing rate of dielectric breakdown strength compared to the control sample**	**Year [Ref]**
Mica	aramid fibrid/CNF	30 wt%	43.39	2018[17]
BNNS	PANF-BNNS nanocomposites	10 wt%	3.91%	2019[9]
NTS	NTS/ANF nanopapers	40 wt%	72.50%	2019[8]
BNNS	PVDF/BNNS nanocomposite films	10 wt%	54.36%	2019[18]
Mica	aramid fibrid	30 wt%	17.31%	2020[19]
Mica	Cellulose nanofibers	50 wt%	74.49%	2020[19]
MTM	ANF/MTM nanocomposite films	5.0 wt%	21.38%	2020[10]
BNNS	ANF-BNNs composite papers	20 wt%	137.45%	2020[20]
RNs	ANF-RNs nanopapers	20 wt%	99.00%	2022[12]
HAP	HAP/ANF nanocomposite papers	20 wt%	21.38%	2022[13]
HNs	hydroxyethyl cellulose	20 wt%	32.29%	2022[21]
Mica	ANF-PEI-Mica nanopapers	50 wt%	74.49%	2023[16]
BSNs	ANF-BSNs nanopapers	0.1 wt%	27.57%	**This work**
		0.5 wt%	68.72%	
		1.0 wt%	132.98%	
		3.0 wt%	162.14%	
		5.0 wt%	204.13%	

Note: CNF-cellulose nanofibers; BNNS-boron nitride nanosheets; NTS-sodium tetrasilicic mica; MTM- montmorillonite; RNs-rectorite nanosheets; HAP- hydroxyapatite; HNs-hydroxyapatite nanowires

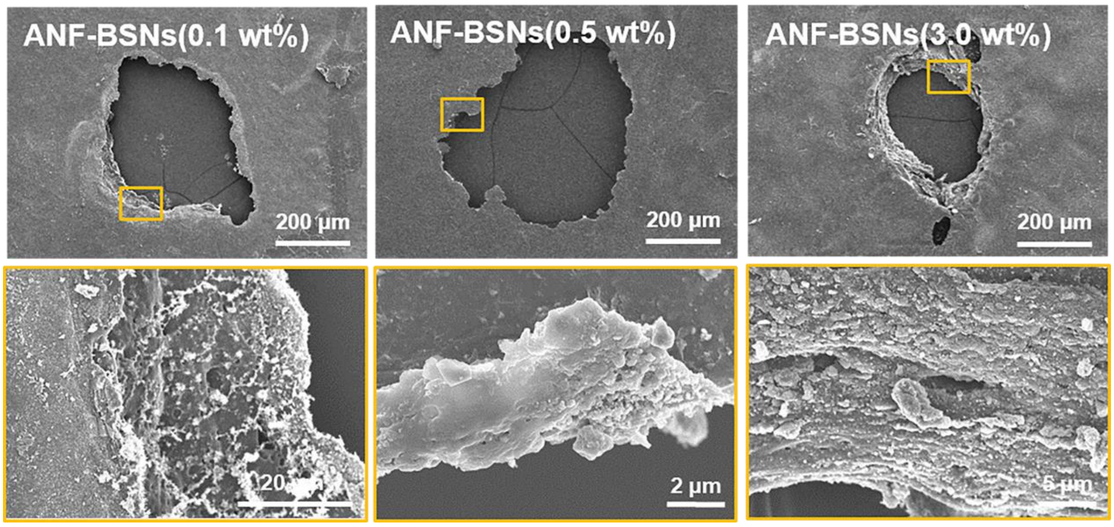

Figure S11. SEM images of dielectric breakdown sites of ANF-BSNs composite nanopapers with different BSNs contents (0.1 wt%, 0.5 wt%, and 3.0 wt%).

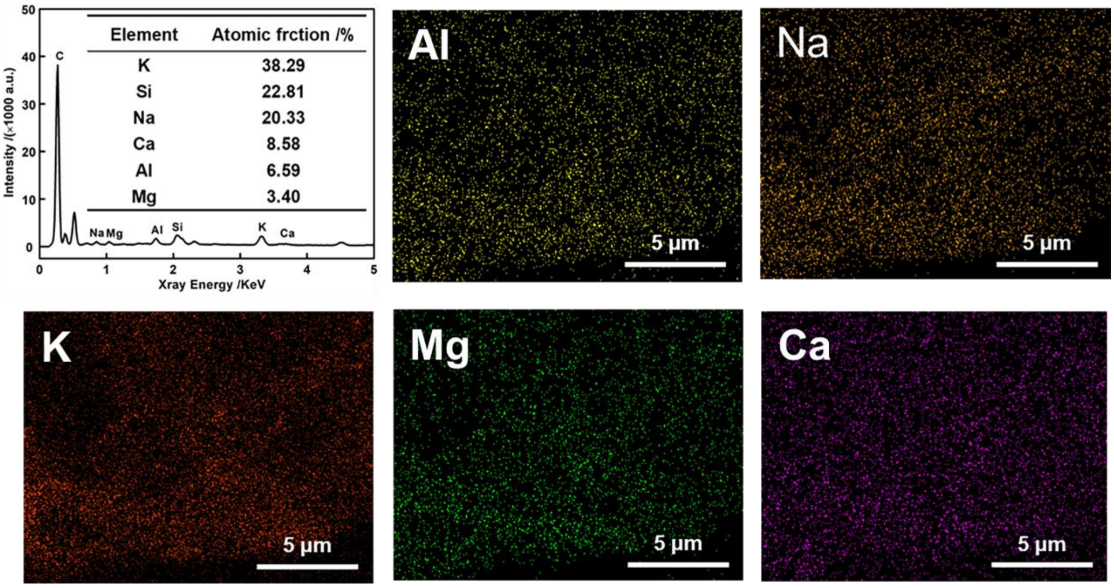

Figure S12. Energy-dispersive X-ray (EDX) image of dielectric breakdown sites in ANF-BSNs composite nanopapers with 1.0 wt% BSNs and its EDX elemental mappings of Al, Na, K, Mg, and Ca in the dielectric breakdown hole surface.

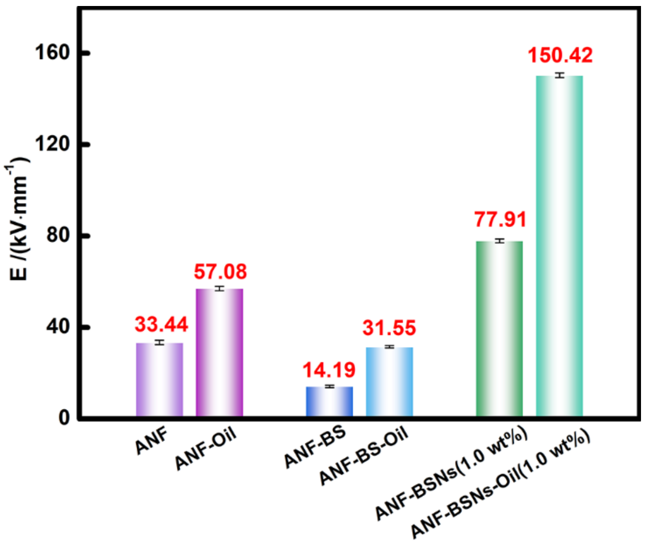

Figure S13. Dielectric strength of ANF nanopapers, ANF-BS micropapers, and ANF-BSNs composite nanopapers in air atmosphere and 150 °C silicone oil bath.

References

[1] T. Yamashita, P. Hayes, *Appl. Surf. Sci.* **2008**, *254*, 2441.

[2] Z. M. Han, D. H. Li, H. B. Yang, Y. X. Zhao, C. H. Yin, K. P. Yang, S. H. Yu, *Adv. Funct. Mater*. **2022**, *32*, 2202221.

[3] S. W. Zhang, Y. X. Yao, H. F. Gao, L. L. Ren, *Metrol. Sci. Technol*, **2021**, *6*, 40.

[4] M. Takayanagi, T. Katayose, *J. Polym. Sci.* **1981**, *19*, 1133

[5] B. Yang, L. Wang, M. Zhang, J. Luo, X. Ding, *ACS Nano*. **2019**, *13*, 7886.

[6] H. J. Chen, Q. Y. Bai, M. C. Liu, G. Wu, Y. Z. Wang, *Green. Chem*. **2021**, *23*, 7646.

[7] S. Ifuku, H. Maeta, H. Izawa, M. Morimoto, H. Saimoto, *RSC. Adv*. **2014**, *4*, 40377.

[8] F. Zeng, X. Chen, G. Xiao, H. Li, S. Xia, J. Wang, *ACS Nano*. **2019**, *14*, 611.

[9] M. M. Rahman, A. B. Puthirath, A. Adumbumkulath, T. Tsafack, H. Robatjazi, M. Barnes, P. M. Ajayan, *Adv. Funct. Mater*. **2019**, *29*, 1900056.

[10] L. Si, Z. Lu, C. Yao, Q. Ma, Y. Zhao, Y. Wang, D. Wang, Z. Jin, *J. Mater. Sci.* **2020**, *55*, 5948.

[11] H. Guo, Y. Li, Y. Ji, Y. Chen, K. Liu, B. Shen, S. He, G. Duan, J. Han, S. Jiang, *Compos. Commun*. **2021**, *27*, 100879.

[12] X. F. Pan, G. H. Yu, H. L. Gao, Z. Z. Wang, Z. Bao, X. Li, S. H. Yu, *Adv. Mater*. **2022**, *34*, 2206855.

[13] Z. Y. Wang, Y. J. Zhu, Y. Q. Chen, H. P. Yu, Z. C. Xiong, *Chem. Eng. J*. **2022**, *444*, 136470.

[14] L. H. Zhao, L. Wang, Y. F. Jin, J. W. Ren, Z. Wang, L. C. Jia, *Compos. Part B Eng*. **2022**, 229, 109454.

[15] L. Zhao, C. Wei, J. Ren, Y. Li, J. Zheng, L. Jia, Z. Wang, S. Jia, *Ind. Eng. Chem. Res*. **2022**, 61, 8881.

[16] X. F. Pan, Z. Bao, W. Xu, H. L. Gao, B. Wu, Y. Zhu, G. H. Yu, J. C, S. C. Zhang, L. Li, H. A. Wu, X. Li, S. H. Yu, *Adv. Funct. Mater*. **2023**, *33*, 2210901.

[17] Y. Zhao, W. Dang, L. Si, Z. Lu, *Cellulose*. **2018**, *26*, 2035.

[18] J. Chen, X. Huang, B. Sun, P. Jiang, *ACS Nano*. **2019**, *13*, 337.

[19] Z. Lu, D. Ning, W. Dang, D. Wang, F. Jia, J. Li, *Cellulose*. **2020**, *27*, 8027.

[20] M. Li, Y. Zhu, C. Teng, *Compos. Commun.* **2020**, *21*, 100370.

[21] J. Huang, S. E, L. Si, J. Li, Z. Tian, Z. Lu, *J. Wood. Chem. Technol.* **2022**, *42*, 15.
